# Seasonal Variation in Birth Rates: Physiology versus Family Planning

**DOI:** 10.1007/s10508-024-03008-y

**Published:** 2024-10-24

**Authors:** Joseph L. Tomkins, Robert Black, Wade N. Hazel

**Affiliations:** 1 Centre for Evolutionary Biology, School of Biological Sciences, The University of Western Australia, Crawley, WA 6009 Australia; 2 School of Biological Sciences, The University of Western Australia, Crawley, WA Australia; 3 Department of Biology, DePauw University, Greencastle, IN USA

**Keywords:** Coital frequency, Birth rate, Twins, Season, Fecundability, Fertility

## Abstract

Seasonal variation in birth rates is a ubiquitous property of human populations. Although the general birth rate is dependent on relatively few parameters—sexual behavior, zygote survival, and the number of ova released—the quantitative influence of the effects of each is difficult to estimate. Research has been directed more towards seasonal variation in physiology and less towards behavioral effects such as preferences for birthing in particular seasons. This may be because alongside seasonal variation in birth rate there is also seasonal variation in the multiple birth rate, which necessarily depends on the same physiological factors as the birth rate, suggestive of a link. Here we use a simulation that follows females through their reproductive lives, replicating the incidence of singleton and twin births that arise from single and double ovulations. We use the simulation to quantify the impact of seasonal variation in the physiological variables that affect birth rate. We can generate the seasonal variation in twinning rates observed in European populations, but this does not generate the magnitude of the observed seasonal variation in birth rate. Our simulation shows that, despite correlations between twinning rate and general birth rate, seasonal changes in sexual behavior are necessary to explain the magnitude of the seasonal variation in the general birth rate in humans.

**Supplementary Information:**

The online version contains supplementary material available at 10.1007/s10508-024-03008-y.

## Introduction

A population’s general birth rate, defined as the number of births, divided by the number of women between menarche and menopause, informs of historic patterns of investment in offspring as well as predicting future changes in population structure. There are only three factors that affect the general birth rate: (1) the frequency of coitus within the fertile period, (2) the number of ova available for fertilization, and (3) the probability of the conceptus surviving to birth (Basso et al., 1995; Fellman & Eriksson, 2000; James, 1980; Tong & Short, 1998; Tong et al., 1997). The challenge for understanding variation in the general birth rate is that these factors are almost entirely cryptic, rendering an understanding of how behavior and physiology interact to affect demographic change difficult (Weinberg, 2020). Here we take a simulation approach to identifying the importance of each of these factors in determining intra population variation in the general birth rate.

One avenue for understanding the relative importance of behavioral and physiological effects on the general birth rate is by examining temporal changes within populations. Indeed, there is a consistent pattern across human populations of seasonal variation in birth rate that has attracted the attention of those interested in the underlying causes of variation in the general birth rate (Calot & Blayo, 1982; Eriksson et al., 1988; Lam & Miron, 1987, 1991a, 1991b, 1994; Lam et al., 1994; Martinez-Bakker et al., 2014; Russell et al., 1993). These seasonal patterns vary on large geographic scales (Calot & Blayo, 1982; Lam & Miron, 1994) and can also change subtly through time (James, 1990; Martinez-Bakker et al., 2014).

Alongside the seasonal variation in birth rate, there is also well-documented seasonal variation in the twinning rate (Bonnelykke et al., 1987; Eriksson et al., 1988; Fellman & Eriksson, 2000; Imaizumi, 1980; James, 1990; Knox & Morley, 1960; Nonaka et al., 1993). The twinning rate reflects changes in the frequency of double ovulations, but less obviously, also covaries with the probability that any conception will result in a live birth. For example, Atkinson (1986) showed how the probability of a live birth, is related to the frequency of double ovulations which determines the twinning rate, where according to Atkinson (1986) the probability of live birth is a function of two variables, the probability of live birth per zygote (*p*) and the probability that double ovulation occurs (*F*). If double ovulation and the probability of live birth are independent, and the survival of zygotes from double ovulations are independent of one another, then the probability of live birth per cycle is *p*(1 − *F*) + *p*^2^ *F* + 2*p*(1 − *p*)*F*, which is the sum of the probabilities of a singleton live birth from single ovulation, live born twins from a double ovulation, and also live born singletons arising from double ovulations. In this way double ovulation can increase the birth rate.

The dizygotic twinning rate (henceforth ‘twinning rate’, measured as twins per thousand births, with births summed by month) is itself also a function of the probability of live birth per zygote (*p*) and the probability that double ovulation occurs (*F*), so that the twinning rate equals *p*^2^ *F*/(*p*(1 − *F*) + *p*^2^ *F* + 2*p*(1 − *p*)*F*), i.e. the probability of twins divided by the sum of the probabilities of all live births. That twinning rate varies seasonally is evidence that the probability of live birth per zygote (*p*) and/or the double ovulation rate vary independent of coital frequency. This implicates these factors in the observed variation in the general birth rate (Tong & Short, 1998), and raises the question as to whether seasonal variation in these parameters affect the general birth rate. Indeed, seasonal variation in the general birth rate and twinning rate covary in Switzerland with maxima in the spring (March–May) and minima in late autumn (October–December) (Eriksson & Fellman, 2000), and in the USA (Kallan & Udry, 1989). Since the birth rate for singletons and twins both depends on the probability of zygote survival to live birth and the probability of double ovulation per cycle, seasonal variation in either (or both) of these variables could be responsible for this covariance. Alternatively, the physiological variability that drives the variation in twin birth rate may be coincident but not causal, and behavioral variation may actually be responsible for seasonal variation in the general birth rate.

In Europe, summer is the preferred time for starting a pregnancy (Basso et al., 1995) and behavioral variation arising from planning sexual activity in relation to birth season could explain the sinusoidal seasonal variation in the general birth rate (James, 1971a, 1971b, 1990). Indeed, more recently, the planned pattern of births has changed with couples of proven fertility increasingly favouring having a child that is older for its school year (Dahlberg & Andersson, 2019). Against this, seasonal anomalies point towards the extent to which behavioral changes can be detectable, with high general birth rates documented in September in the USA (James, 1990) and in October in Scotland (Russell et al., 1993) that associate with the holidays of Thanksgiving, and Christmas and New Year, respectively. To what extent seasonal patterns in the general birth rate reflect behavioral decisions, or alternatively are part of an underlying physiological seasonal fluctuation remains largely unknown.

Detailed analysis of fertile couples known to be trying to conceive in North America and Denmark shows that attempts at pregnancy peaked in September but that the highest fecundability (physiological effects on conception) was in the late autumn and early winter (Wesselink et al., 2020). Thus, there is evidence for both seasonal variation in behavior as well as seasonal variation in the outcome of attempts at pregnancy. For the most part, evidence of birth season preferences appear to be sparse in the literature and because they require surveys are more difficult to gather than, for example, environmental data (Basso et al., 1995; Regnier-Loilier, 2010; Wesselink et al., 2020). This knowledge gap may be important, because many of the observed patterns, such as latitudinal variation in the amplitude of seasonal effects could reflect historical adaptive parental decisions to avoid harsh environmental conditions that may no longer apply and preference information cannot be readily gleaned from historical records.

Here we quantify the extent to which seasonal variation in the double ovulation rate and per zygote survival can explain the pattern of seasonal variation in the general birth rate. If variation in these factors is unable to explain the seasonal pattern of variation in general birth rate, then other factors, such as seasonal variation in sexual behavior, are necessarily implicated to be responsible. We can do this because unlike previous studies we are able to simulate both, how polyovulation and per zygote survival directly affect twinning rates and the general birth rate and simulated how “behavioral” changes in the numbers of women exposed to the “risk of pregnancy” also influence these patterns. The cryptic nature of all three of variables that alter birth rate makes a simulation approach an insightful tool.

## Method

This research is a mathematical simulation supported by data already available from the literature and does not require approval from any institutional review boards.

*Population samples* We retrieved the relative changes (‘index counts’) in numbers of births per month (Supplementary Methods) for 23 European countries from Calot and Blayo (1982) (Table 1). To analyze changes in the seasonal shifts across these populations, we generated a “*seasonal bias’*” parameter, which weighs births early in the year negatively and those later in the year positively (Supplementary Methods).

**Table 1:**
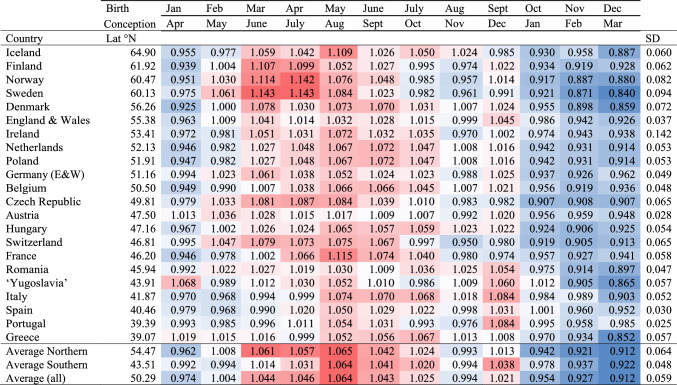
Seasonality of birth and conception indexed to a mean of one with their standard deviation, for 22 European countries ranked by latitude (Lat)

Within each population, intensity of red indicates elevated general birth rates, intensity of blue depressed general birth rates. Populations Iceland to Hungary were deemed “Northern”. Data from Calot and Blayo (1982)

We retrieved data from the literature for raw numbers of births by season in population samples from Sweden 1973 (Sandahl, 1978), England and Wales 1952–1975 (James, 1980) and Scotland 1938–1987 (Russell et al., 1993) (Table 2A, B).

**Table 2:** (A) Numbers of births per month for sample cohorts from three European populations. Numbers of births are adjusted for variation in the number of days in the month. (B) Number of births indexed to a mean of one, showing the variability across the year. Swedish data is most variable, Scottish least variable

Birth month	Scotland	England and Wales	Sweden
(*A*)* Adjusted numbers of births*			
January	369,918	1,339,280	7582
February	368,593	1,264,353	8379
March	380,650	1,443,853	8636
April	381,720	1,352,945	9124
May	382,930	1,400,164	8526
June	375,261	1,322,845	8115
July	370,834	1,344,632	7702
August	358,462	1,302,065	7419
September	352,838	1,293,074	7648
October	372,662	1,280,561	7275
November	341,854	1,203,090	6773
December	348,677	1,258,625	6707
(*B*)* Index numbers of births*			
January	1.008	1.017	.970
February	1.004	.960	1.072
March	1.037	1.096	1.105
April	1.040	1.027	1.167
May	1.043	1.063	1.091
June	1.022	1.004	1.038
July	1.010	1.021	.985
August	.977	.989	.949
September	.961	.982	.978
October	1.015	.972	.931
November	.931	.913	.866
December	.950	.956	.858
SD	.0370	.0496	.0957

James (1990), Russell et al., (1993), Sandahl (1978)

We retrieved monthly twinning rate data for Switzerland 1876–1930 (Eriksson & Fellman, 2000), Denmark 1855–69, (Fellman & Eriksson, 1999) and England and Wales 1952–1975 (James, 1980) (Table 3A, B).

**Table 3:** Monthly variation in the (A) twinning rates calculated as twins per thousand births and (B) the relative (indexed) twinning rates, of three European populations

A	Birth	Switzerland	England and Wales	Denmark	
Conception		Observed	Observed	Observed	Expected
May	January	12.46	7.87	14.00	14.65
June	February	12.87	7.74	14.50	15.07
July	March	13.26	7.69	15.90	15.32
August	April	13.22	7.54	16.20	15.35
September	May	13.21	7.36	15.00	15.20
October	June	13.20	7.51	14.80	14.85
November	July	12.40	7.41	14.00	14.37
December	August	12.43	7.58	13.40	13.96
January	September	11.92	7.79	13.90	13.65
February	October	12.00	7.59	14.30	13.65
March	November	12.35	7.71	14.25	13.80
April	December	12.53	7.69	13.92	14.20

B	Birth	Relative twinning rates		Denmark	
		Switzerland	England and Wales		
Conception		Observed	Observed	Observed	Expected
May	January	.985	1.033	.965	1.010
June	February	1.017	1.016	.999	1.039
July	March	1.048	1.009	1.095	1.056
August	April	1.045	.989	1.116	1.058
September	May	1.044	.965	1.033	1.048
October	June	1.043	.985	1.020	1.024
November	July	.980	.972	.965	.991
December	August	.982	.994	.923	.962
January	September	.942	1.022	.958	.941
February	October	.948	.995	.985	.941
March	November	.976	1.012	.982	.951
April	December	.990	1.009	.959	.979
	SD	.0382	.0203	.0576	.0450

Eriksson and Fellman (2000), Fellman and Eriksson (1999), James (1980)

We used the Calot and Blayo (1982) data to get an overall picture of how birth rates vary seasonally and latitudinally across Europe. We used the remaining data on age dependent and seasonal variation in twinning to see if the variables that influence twinning rates are responsible for the seasonal covariance in twinning and birth rates, or whether changes in behavior are also implicated.

*Simulation model* We used simulations to reproduce the pattern of seasonal variation in twinning rates by altering probability of live birth per zygote and rate of double ovulation. Further details of the simulation model are given in Hazel et al. (2020). The model is run in R (R Core Team, 2021) and simulates the reproductive lives of *n* women subject to a series of events (early-, mid-term-, late-loss, birth, weaning… etc.) that are executed on the basis of probabilities taken from the literature. Each event is associated with time penalties derived from the literature, eventually leading to a return to ovulatory cycling and the possibility of conception or an age-dependent menopause. There are no social factors at play and there is no predetermined variation in female “quality” that might shape any of the probabilities or parameters in this simulation. [Supplementary Methods and Hazel et al. (2020)].

**Fig. 1:**
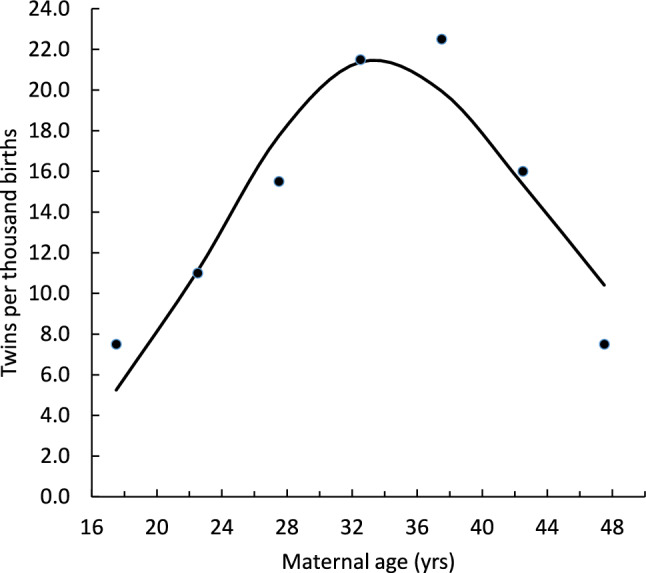
Age and twinning frequency for a population sampled from Denmark 1921–1930 (Fellman & Eriksson, 2005). The line represents the best fit of a model in which there is an age-dependent switch to double ovulation and declining probability of live birth per ova (Hazel et al., 2020)

In the simulation, women start reproduction at age 18 with a given probability of live birth per ova, which declines exponentially through their lives. This *decline rate* is characterized by an intercept at age 18 (*dra*) and a slope (decline rate) (*drb*) that determines the year-on-year % decrease with age. As they age, the probability of releasing two ova in any given cycle increases as a cumulative normal function with pre-set mean (*spm*) and standard deviation (*spSD*). Hazel et al. (2020) modelled a cumulative normal distribution, because the switch from single to double ovulation represents a conditional evolutionarily stable strategy in which variation in age of switching is normally distributed, as any female ages they are more and more likely to have switched to double ovulation. The probability of live birth per zygote in women at age 18 years (*dra*) sets the female’s trajectory for this aspect of reproductive physiology for life. The age at which the average female switches to double ovulation (*spm)* governs the rate of double ovulations in the population. We used datasets for the age-dependent twinning rates from 1921 to 1930 for Denmark from (Fellman & Eriksson, 2005) [their Figure 6 and the formulae of Hazel et al. (2020)] to establish baseline values for *dra* = .550, *drb* = .898, *spm* = 39.8 and *spSD* 9.44 which give rise to the fit shown in Fig. 1.

*Seasonal twinning rates* We attempted to replicate Fellman and Eriksson’s (1999) expected (i.e. generated from their seasonal model fit) seasonal variation in twinning rates for Denmark (1855–1869) by individually varying the simulation’s parameters (*dra* and *spm*). These data are ideal because they include a large amplitude in twinning rates (see Fig. 1 in Fellman and Eriksson [1999]). Thus, either the probability of double ovulation (*spm*) or the probability of zygote survival (*dra*) must vary dramatically across the season in this population. If these physiological factors alone are enough to recreate the seasonal variation in twinning rate, then the general birth rate should also be affected. If it is not affected to the degree observed, then behavioral factors are implicated in causing the remaining seasonal variation in the general birth rate. We determined the twinning rates produced by a range of values for *dra* and *spm* in separate sets of simulations for each variable and regressed these twinning rates on *dra* or *spm* (Figure S1 and S2) to estimate the sequence of values of *dra* or *spm* needed to obtain the seasonal pattern of twinning rates seen in the Danish data.

Once we had established the sequential seasonal changes in *dra* and *spm* needed to shift twinning rates in the seasonal pattern, we ran simulations that replicated each month’s value of *dra* or *spm* 15 times for 1000 individuals. This therefore simulates a population living their entire reproductive lives under the *dra* or *spm* value we specified for each month. In this way we keep the physiological effects on the probability of live birth arising from an ovulatory cycle, separate from the effects of the size of the cohort of women sexually active at the time of ovulation (Supporting Methods). From the 180 runs of the simulation (15 times for each month), we were able to record the number of twins born per thousand births and the average lifetime number of births. We plotted a loess fit to the seasonal variation in twinning rates data and overlaid the data from the fitted values of Fellman and Eriksson (1999).

*Seasonal variation in the number of births* Here we used a 1938–1987 data set from Scotland (Russell et al., 1993) to understand how the numbers of births change seasonally. These data are particularly valuable because they are (1) a large sample, (2) have raw numbers of births, (3) rather than relying on the observed values, data are accompanied by expected numbers of births for each month derived from a cosinor analysis, and (4) when compared with other data sets, represent a modest amount of seasonal variation [SD = .037 vs .048 and .059 for “outhern” and “all” European populations, respectively (Table 2)]. Hence the seasonal variation in the general birth rate in this population ought to be relatively easy to account for by changing our model parameters and therefore represent a conservative data set to test against. We used the expected (smoothed) values as well as the raw values to estimate expected monthly birth variation. We used the smoothed value from Russell et al. (1993) that removes the October anomaly that arises from conceptions around Christmas and New year.

*Can simulated seasonal variation in twinning rates yield seasonal variation in birth rates?* To estimate the effect of our simulated variation in twinning rates on general birth rate we first asked whether the seasonal variation in twinning rates yielded a seasonal pattern in the number of births. This requires the variation in general birth rate to be standardized such that we are analysing departures from an average number of births across the year. However, this only shows the similarity of pattern not the magnitude: to determine whether the variation in the amplitude of the seasonal variation in general birth rate could be explained by our manipulations of *dra* and *spm* alone, we transformed the Scottish data following (Fellman & Eriksson, 1999) by dividing by the average of the 12-month general birth rate. This produces a data set with a mean of one but retains the amplitude of the seasonal variation across the year. To compare this seasonal variation with the simulations we converted the average number of parities per female into a total number of births by multiplying the monthly per capita general birth rate by the number of individuals in the simulation (1000). We divided this value by the average across the 12 months to get a mean of one but retaining the variation in proportion to the mean of one.

Because we were unable to explain all of the seasonal variation in birth rate using the variables that affect twinning rates (*dra* and *spm*) we asked how much the size of the at-risk cohort of women (women sexually active at the time of ovulation) would have to change to match the variation in the numbers of births across the year, we used the output from the simulations of *dra* and *spm* and their effects on the total numbers of births to predict the variation in the number of individuals needed to meet the fluctuation in births per month, over and above that which could be explained by our simulated variation in *dra* or *spm*. We report the variation expressed as a percentage (Supplementary Methods).

**Fig. 2:**
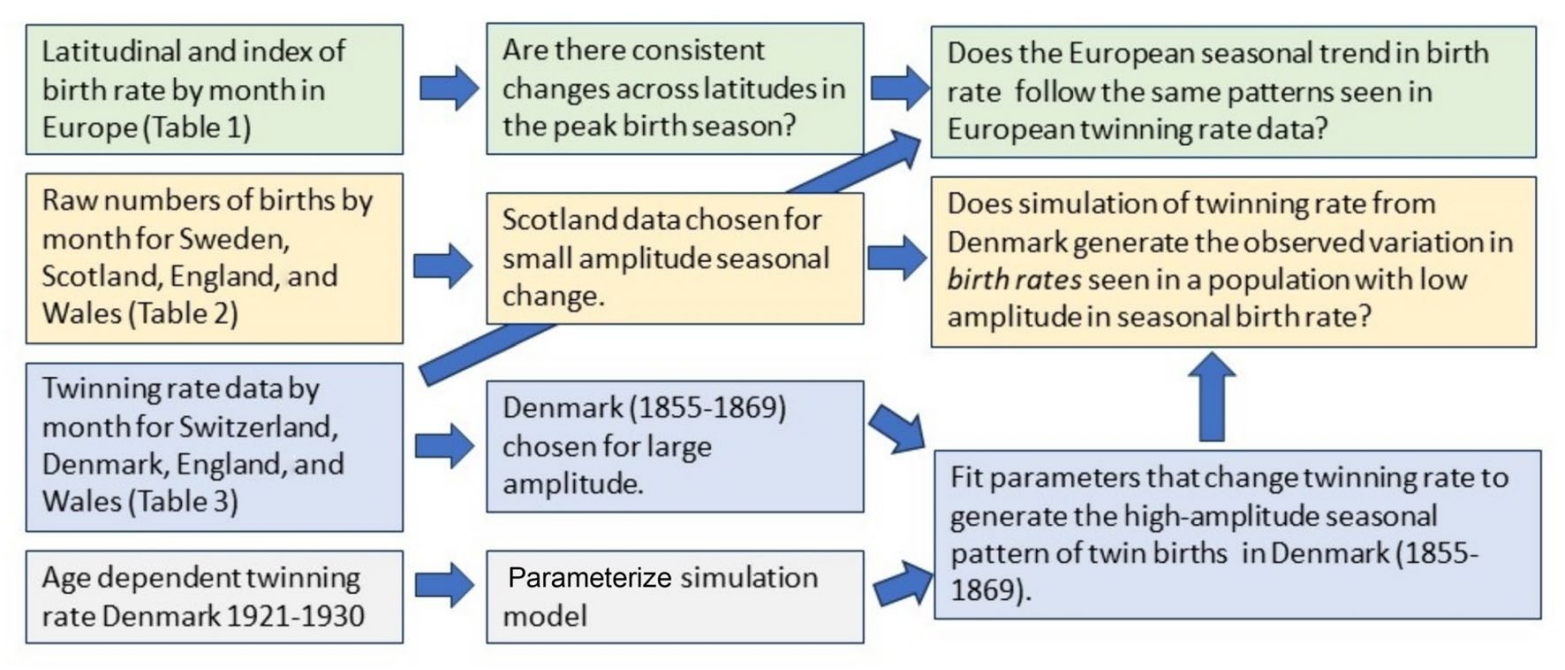
Methodological structure

For a graphical representation of the methodology applied to each data set, see Fig. 2.

## Results

The seasonal variation in the general birth rate for Europe (Table 2) was characterized by a northward increase in seasonal variation (estimated as the standard deviation of the index births per month) (Pearson’s correlation coefficient *t*_20_ = 2.18, *r* = .44, *p* = .038). The months of peak births were from March to May, with this “seasonal bias” shifting to be later in the year in more southerly countries (Table 3, *t*_20_ = − 2.60, *r* = − .50, *p* = .017).

**Fig. 3:**
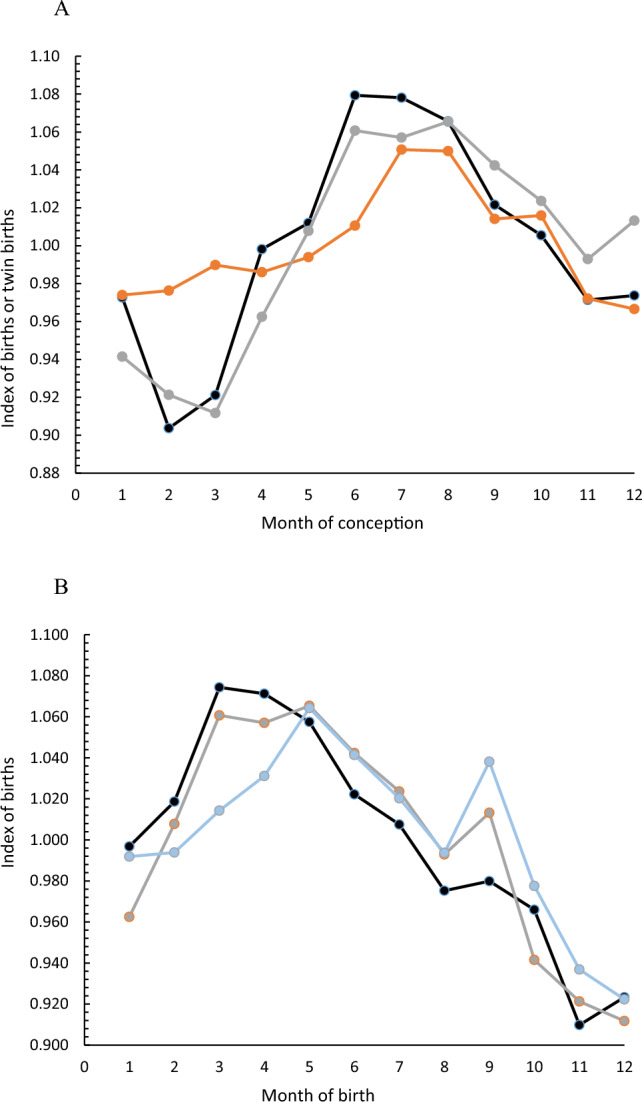
**A** Index number of births of the focal populations (black line) and Calot and Blayo (1982) northern European data set (grey line) and the index of twinning rates for focal populations (orange line) against month of conception. **B** Index number of births focal populations (black line) Calot and Blayo (1982) northern European data (grey line) and Calot and Blayo (1982) southern European data (blue line) against month of birth, showing the September anomally (Color figure online)

Seasonal twinning rates, derived from the populations in Table 3, and the general birth rates of Northern European populations (Tables 1, 2) show a similar seasonal rise and fall (Fig. 3A). Both sources of European data show the effect of excess conceptions in December/January that give rise to the September spike in births (Fig. 3B).

**Fig. 4:**
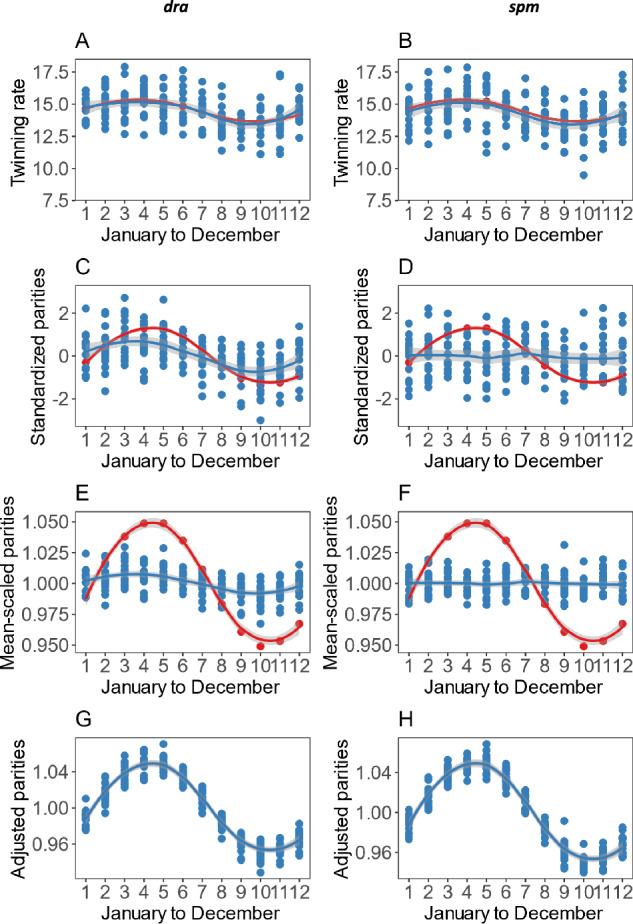
Patterns of seasonal variation in twinning rate and number of parities (red lines and points) alongside simulations (blue lines and points, with grey standard error to a Loess fit) varying the probability of live birth per ova with season (left panel) and varying the age-dependent switch to double ovulation (right panel). **A** Model fit to observed seasonal twinning rates from Denmark (Fellman & Eriksson, 2005) with 15 simulations per month varying the probability of live birth per ova across the year and **B** varying the age at the switching to double ovulation. **C** Changes in fecundity with season in Scotland (Russell et al., 1993) standardized to have mean of zero and standard deviation of one (red line) alongside the similarly standardized number of parities produced from the simulation when probability of live birth is varied and **D** when age at switching to double ovulation is varied. **E** Data from **C** with scaled mean, but the standard deviation allowed to vary, showing how although the pattern is the same as in **C**, the amplitude is reduced in the simulated data set. **F** Data from **D** with scaled mean, but the standard deviation allowed to vary, showing only poor concordance. **G** Data from **E** with additional seasonal variation in the size of the at-risk population. **H** Data from **F** with additional seasonal variation in the size of the at-risk population (Color figure online)

Using the simulation model (Hazel et al., 2020) and applying variation only to the probability of live birth per zygote (*dra*), in a manner that rose and fell with the seasonal patterns of twin births from 1921 to 1930 in Denmark, we were able to simulate a seasonal relationship that produced the observed twinning rates in Denmark, by varying *dra* by 10.72% in a range between 0.66 and 0.73 (Fig. 4A). Similarly, we were also able to recreate the variation in twinning rates across the year by manipulating the mean of the age-dependent switch from a single to double ovulation (*spm*) by 2.13%, between 37.18 and 37.98 (Fig. 4B) (the large standard deviation of the *spm* means that although the switch seems ‘late’ many women still switch to double ovulation before menopause). Both changes in *dra* and *spm* will affect the birth rate of the population, with more parities when *spm* is low (women switch at a younger age) and more parities when *dra*, the probability of live birth per ova is elevated. It is difficult to compare the variation in *spm* and *dra* required to achieve the variation in twinning rates because they are on different scales, probability vs age, respectively. However, while seasonal changes in both *dra* and *spm* could account for the observed seasonal fluctuations in twinning rates, only fluctuations in *dra* yields a seasonal change in general birth rates consistent with those seen in Scotland—a typical northern European population (Fig. 4C, D).

Manipulating *dra* to match the twinning rates, replicates the pattern of rise and fall in the total general birth rate; however, the amplitude of the variation in births across the year is very much less if only *dra* is altered to satisfy the twinning rate data (Fig. 4E, F). Thus, although seasonal variation in *dra* can explain the seasonal variation in twinning rates, it only explains some of the seasonal variation in general birth rates. Hence, other explanations beyond variation in probability of live birth per zygote are needed to fully explain seasonal changes in the general birth rate.

We were able to explain the remaining variation in general birth rate across the year by simulating variation in the number of women at risk of becoming pregnant. For the simulation of *dra* to replicate the observed seasonality in births (in addition to the aforementioned 10.72% change in *dra*) a change of 8.4% (1044–960; Fig. 4G) was required in the size of the pool of women at risk of pregnancy. For the simulation of *spm* (and in addition to the 2.12% change in *spm*) a 10.0% (1051–951) change in the size of the pool of at-risk women was required across the year (Fig. 4H). Because this is a simulation the exact numbers are different for each set of runs because each run returns different values; however, the values we report here for the 180 simulations were consistent across numerous runs.

## Discussion

The co-variation between twinning rates and general birth rates suggests that factors that affect both, specifically probability of live birth per zygote and double ovulation rate, might explain the seasonal patterns in the general birth rate (Fellman & Eriksson, 2000; Lam & Miron, 1994). However, our analysis suggests that such factors cannot explain all the seasonal variation in birth rates. Subtle variation in the proportion of women exposed to the risk of pregnancy at the time of ovulation exerts enough influence on seasonal variation in general birth rate, to supplement seasonal variation due to physiological changes in per capita live birth rate or double ovulation rate so that our simulations match empirical patterns.

It is plausible that the rate of double ovulation is critical to the co-variation between twinning rates and the general birth rate (Eriksson & Fellman, 2000; James, 1990). However, we show, that although the frequency of double ovulations in the population can indeed replicate changes in twinning rates across the year, it has a very limited effect on the overall number of births (Fig. 4D, F). There are three reasons why this effect is weak: first, the probability of a twin birth arising from a double ovulation is dependent on the square of the probability of live birth per zygote (Atkinson, 1986; Hazel et al., 2020). Second, the vast majority of double ovulations occur in older women who, all things being equal, make a relatively small contribution to overall numbers of births because as women age, the probability of live birth per zygote decreases. Further to this age effect, our simulated population is conservative in the sense that there are no social influences at play that might normally reduce the likelihood of older women attempting pregnancy, e.g. having achieved their target family size.

Increasing the probability of live birth per zygote (*dra*) has a greater influence the seasonal patterns in general birth rate (Fig. 4A vs B) because it affects women of all ages. Unlike other studies, we could quantify the magnitude of the variation in the probability of live birth per zygote that is required to generate the seasonal pattern of age-dependent twinning observed in a typical European (Danish) population. This shows that the variation in the twinning rate can be explained by a 10% change in the probability of live birth per zygote, but that this is insufficient to explain the variation in the birth rate. This suggests reasonably subtle variation in the probability of live birth per zygote can explain some seasonal variation in birth rate, even when the double ovulation rate, which has been considered the more obvious candidate for the seasonal variation (Eriksson & Fellman, 2000; James, 1990), is held constant across the year. The co-occurrence of seasonal variation in twin and triplet births is also consistent with changes in probability of live birth per zygote, since with a constant frequency of double or triple ovulations, seasonal variation in the probability any zygote surviving to birth will cause twin and triplet rates to covary across the year.

We did not simultaneously manipulate *dra* and *spm*. However, the independent manipulation of each shows that both can affect twinning rates, but the effects of *spm* are much less predictable than those of *dra*. Thus, if *spm* covaried in a way that elevated double ovulations at times of year when zygote survival was highest, any simultaneous manipulations would inevitably indicate that smaller changes in *dra* are necessary to achieve a fit to twinning rate. Hence the independent manipulations are conservative.

By knowing the population size in the simulation and the amplitude of the seasonal variation needed to replicate the observed twinning rates, we can explicitly determine the variation in the numbers of births that is caused by reproductive physiology alone (*dra* and *spm*) in a numerically stable population. From there, we can estimate how the fraction of sexually active females vary to achieve the observed seasonal variation in births. Even though 10% variability in probability of live birth per zygote is enough to explain the variation in twinning rates, and does generate some seasonal variation in the general birth rate, it is insufficient to account for all of the variation seen in total births, something previously un-resolved (Eriksson & Fellman, 2000; James, 1990). Our simulations show that, in addition to seasonal variation in probability of live birth per zygote, there must also be variation in the fraction of sexually active women (Fig. 4G, H). The change in this fraction required appears to be 10% across the year. This is in line with estimates derived from surveys of 5–12% across six European countries (1970–1973) (Basso et al., 1995), 13% in France (Regnier-Loilier, 2010) and 16% in the USA and Denmark (Wesselink et al., 2020), the latter two being a more recently studied cohorts (2005 and 2007–2018, respectively).

Changes in the numbers of births due to variation in the size of the pool of women at risk of pregnancy is directly evidenced by the anomalously high number of births in September associated with sexual activity over New Year and Christmas (see also James, 1990; Lam & Miron, 1994; Russell et al., 1993) for the European populations documented here (Fig. 3B). There is also a deficit of conceptions in February and March (Fig. 3A) in comparison to that expected from the twinning rate, which is unaffected by behavioral changes. These patterns are known, but highlight how important behavioral changes affecting pregnancy risk are likely to be in the overall generation of seasonal patterns in general birth rate. For example, in February, the physiological determinants of fertility are not as low as the general birth rate would suggest. Planning is likely to favour spring births in the north (of the northern hemisphere) where summers are short and disfavor winter births because winters are harsh. Indeed seasonal variation in the frequency of intercourse in couples trying to get pregnant in modern populations remains evident (Dahlberg & Andersson, 2019), and fertile couples tend to plan their pregnancies even in relation to factors such as the school year (Wesselink et al., 2020). The declining amplitude of the seasonal effects found here and their shifts to later summer births in the south (see also Martinez-Bakker et al., 2014) are consistent with adaptive choices for the avoidance of childbirth during winter (Karlsson, 2018). Presumably, last century and before, agricultural work, seasonal food shortages and the predictable increase in respiratory diseases would have further enhanced preferences for birth season.

Seasonal co-variation in reproductive physiology (probability of zygote survival and double ovulation rate) estimated from twinning and general birth rate could, in principle, be attributable to the choices made by older women (with proven child-bearing ability) about when to have their children. Such a pool of women who are also more likely to bear twins might join the background at-risk population at a time of year that coincides with resources available for a successful pregnancy (Bonnelykke et al., 1987). Our simulation (Fig. 4D) suggests that if there is such an effect of a sub-group of fertile women choosing their birth seasons, the effect is most likely driven by the numbers of women rather than there being a substantial influence of their age and probability of twin bearing, since our manipulation of the age-dependent double ovulation rate yielded a very poor signal when it came to seasonal variation in general birth rate.

The seasonal variation in twinning rate is likely to be a reliable measure of environmental impacts on the physiological basis to fertility (Tong & Short, 1998), while variation in birth rate will more reflect preferences and cultural variation. Peaks in general birth rate should therefore tend to be more variable than peaks in twinning rates among and within populations with different cultural practices. This expectation is exemplified among Muslim and Jewish ethnic groups in Israel where general birth rates are offset, but there is nevertheless a common peak in the twinning rate (Picard et al., 1990). Temporal changes (Martinez-Bakker et al., 2014) and miss-alignment between the general birth rate and higher order birth rates (Elster & Bleyl, 1991; James, 1980) also support this conclusion. Unfortunately, data on twinning rate and general birth rate that have the power to detect seasonal patterns are sparse, and modern twinning rates vary for other reasons such as assisted reproductive technologies (Monden et al., 2021). It would be intriguing to know the seasonal patterns of twin births and general births in tropical climates, but we have been unable to source such data.

## Conclusions

Our simulations suggest that although double ovulation rates and the probability of zygote survival can align seasonally with the general birth rate (Bonnelykke et al., 1987; Eriksson & Fellman, 2000), this correlation does not appear to be indicative of a substantial causal relationship. Rather, double ovulation rates and the probability of zygote survival can vary by at most 10% across the year with only limited effects on the general birth rate. Our simulation quantitatively supports the notion that the factor accounting for most of the seasonal variation in general birth rate must be behavioral (James, 1971a, 1971b, 1990), and likely indicative of substantial preferences for particular birth seasons (Basso et al., 1995; Dahlberg & Andersson, 2019; Wesselink et al., 2020). We conclude this because variation in the physiological determinants of fertility derived from a population with high amplitude seasonal twinning rates, cannot generate the peaks and troughs seen even in a population with a low amplitude in the seasonal variation in the numbers of births. Given the costs associated with childbearing and rearing in humans, the socially monogamous mating system, and the ability to plan and exert behavioral control over the timing of child birth (Basso et al., 1995; Regnier-Loilier, 2010; Wesselink et al., 2020), the conclusion that substantial seasonal variation in general birth rate results from seasonal changes in sexual activity does not seem surprising, but is now well supported by a model that accounts for variation in both physiology and behavior.

## Supplementary Information

Below is the link to the electronic supplementary material.

Supplementary file1 (DOCX 166 KB)

## Data Availability

Data will be available on Dryad 10.5061/dryad.vq83bk3vh.

## Code Availability

Code will be available on Drya.
