# Supplementary material for: Seasonal Variation in Birth Rates: Physiology versus Family Planning
Source: Arch Sex Behav. 2024 Oct 24;54(1):107–16. doi: 10.1007/s10508-024-03008-y (PMC11782445; doi:10.1007/s10508-024-03008-y)
Supplement: Supplementary file 1 — Supplementary file1 (DOCX 166 KB) [file 10508_2024_3008_MOESM1_ESM.docx]

**Supplementary Methods**

*Gestation*: Alignment of twin and singleton conceptions to month was done by assuming twin gestation to be eight months and singleton gestation to be nine. The general birth rate data (Tables 1, 2 and 3) were assumed to be for singletons.

*Population samples*: Index values for the populations in Table 2A are given in Table 2B. To derive the index value, the monthly counts are divided by average monthly count (Table 2) Index values provide a way to compare patterns and variability among populations (Eriksson & Fellman, 2000; Fellman & Eriksson, 1999).

*Seasonal Bias*: This was calculated by multiplying the index number of births in a month by the rank of the birth month (where Jan = 1 and Dec = 12), summing these values and dividing by the sum of the month’s ranks (i.e. 1+2+.. 11+12 = 78).

*Simulation model*: In the original simulation the value for *dra* was 0.55 as this represents the best fit to the most comprehensive data for a natural fertility population (Hazel et al., 2020; O'Connor et al., 1998) and for *drb* is 0.89 which is the average value for the populations analysed in (Hazel et al., 2020). Conceptions are not sperm limited, but there are early, midterm and late losses between ovulation and birth; post birth survival is not considered here. Mothers face mortality risks from births and a constant annual risk of death. Women continue their reproductive cycles subject to these parameters between ±18 and up to 40 years of age.

The simulation has an age structure that has a flatter distribution than might be expected e.g. (Sandahl, 1978) and is slightly biased towards older individuals that will have more twins, and is therefore likely to be an overestimate of the twinning rates of the population rendering our analysis conservative.

Here for the various values of *dra*, (0.66 to 0.735) *spm* was held constant at 39.8, and *spSD* at 9.44 and for the various values of *spm* (37.18 to 37.98), *dra* held was constant at 0.55 and *spSD* at 9.44, these are values of *spm* *spSD* and *dra* that produce the best fit to the observed age dependent pattern of twinning. These sets of 12 values gave twinning rates that matched the 12 Danish rates had a small range for both *dra* and *spm*.

Variation in *dra* and *spm* was expressed as a % and calculated as

$$100*\frac{(max value-\min value)}{average value}$$

The values of *spm* and *dra* we manipulated to match the twinning rate differ from those estimated in Figure 1. This likely reflects the temporal difference between the seasonal data (1855-69) and the age data (1921-30), and the temporal decrease twinning rates seen across Europe (Eriksson & Fellman, 2007; Eriksson et al., 2008; Fellman & Eriksson, 2003). Earlier age at the switch to double ovulation (*spm*) reduces the value of the probability of live birth per ova (*dra)* needed to match the observed twinning rate, while higher probability of live birth per ova increases the age at the switch to double ovulation. Our conclusions are robust to any temporal shift because we are interested in the variability in *dra* and *spm* not their absolute values.

*Size of the at-risk cohort*: Excess (or deficits) of conceptions in one month might reduce (or increase) the size of the at-risk cohort in subsequent months (Lam & Miron, 1994). However, these effects diminish rapidly and are dependent on initial cohort sizes. Furthermore, in addition to this source of variation in the cohort size, there will also be new recruits and others returning to the at-risk population after childbirth or foetal loss as well as those exercising birth season preferences. We include all these effects in our modelling of the variation in the size of the at-risk cohort.

*Seasonal Pattern*: To standardise the seasonal variation the data from Scotland 1938-1987 (Russell et al., 1993) showing variation in general birth rate, and the twinning rates from the simulation were both standardised to a Z score:

$$\frac{x-\overline{x}}{s}$$

Where *s* is the standard deviation of the sample.

*Seasonal variation*: The percentage change in the cohort size needed to match the variation in the numbers of births was calculated as:

$$100*\frac{(max cohort size-\min cohort size)}{average cohort size}$$

**Figure S1
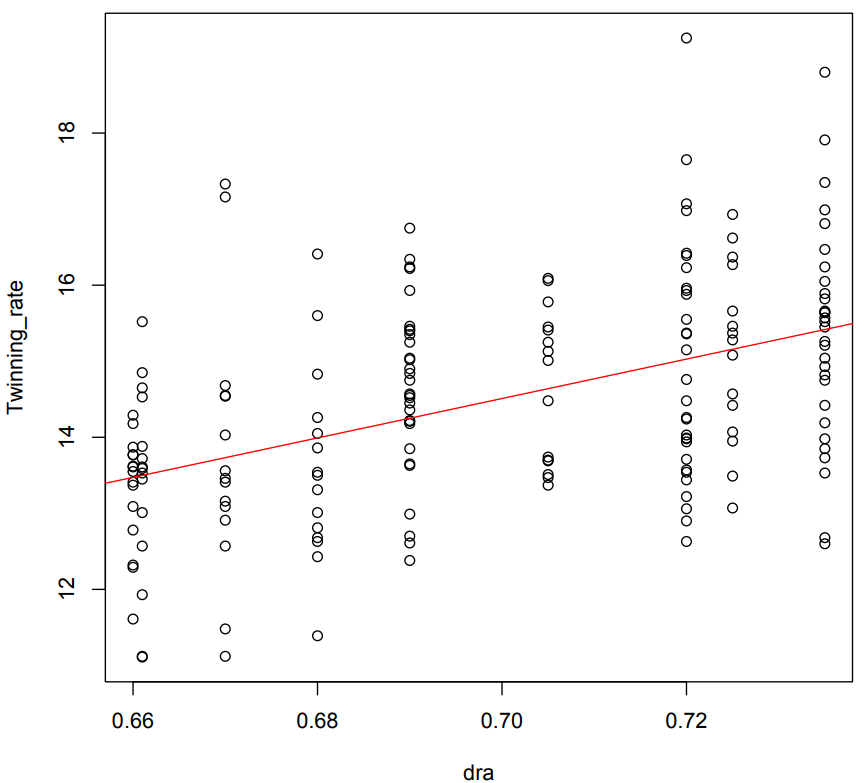
**

**Figure S1.** The relationship between values of *dra* and the twinning rate derived from numerous runs of the simulation. Each datapoint represents one simulation. The regression equation ~ Twinning rate = -3.635+*dra**25.921.

**Figure S2
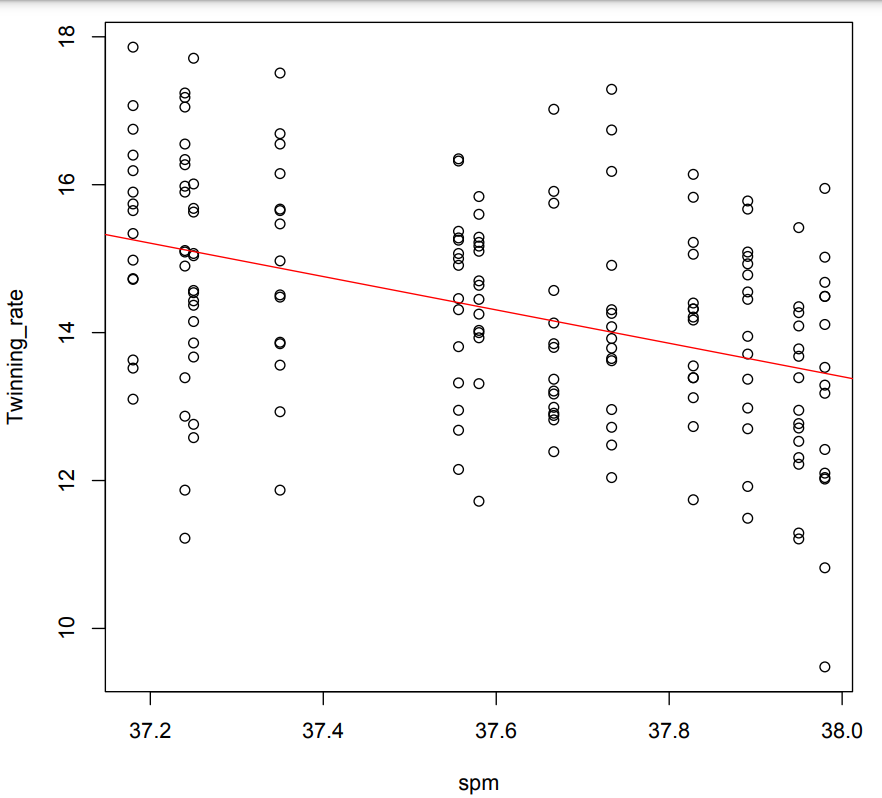
**

**Figure S2.** The relationship between values of *spm* and the twinning rate derived from numerous runs of the simulation. Each datapoint represents one simulation. The regression equation ~ Twinning rate = 99.1027+*spm**-2.552.

**References**

Eriksson, A. W., & Fellman, J. (2000). Seasonal variation in livebirths, stillbirths extramarital births and twin maternities in Switzerland. *Twin Research*, *3*, 189-201.

Eriksson, A. W., & Fellman, J. (2007). Temporal trends in the rates of multiple maternities in England and wales. *Twin Research and Human Genetics*, *10*(4), 626-632. <https://doi.org/10.1375/twin.10.4.626>

Eriksson, A. W., Fellman, J., Jorde, L. B., & Pitkanen, K. (2008). Temporal, seasonal, and regional differences in births and deaths in Aland (Finland). *Human Biology*, *80*(2), 125-140. <https://doi.org/10.3378/1534-6617>

Fellman, J., & Eriksson, A. W. (1999). Statistical analysis of the seasonal variation in the twinning rate. *Twin Research*, *2*, 22-29.

Fellman, J., & Eriksson, A. W. (2003). Temporal differences in the regional twinning rates in Sweden after 1750. *Twin Research*, *6*(3), 183-191. <https://doi.org/10.1375/136905203765693834>

Hazel, W. N., Black, R., Smock, R. C., Sear, R., & Tomkins, J. L. (2020). An age-dependent ovulatory strategy explains the evolution of dizygotic twinning in humans. *Nature Ecology & Evolution*, *4*(7), 987-+. <https://doi.org/10.1038/s41559-020-1173-y>

Lam, D. A., & Miron, J. A. (1994). Global Patterns of Seasonal-Variation in Human-Fertility. *Human Reproductive Ecology*, *709*, 9-28. <https://doi.org/10.1111/j.1749-6632.1994.tb30385.x>

O'Connor, K. A., Holman, D. J., & Wood, J. W. (1998). Declining fecundity and ovarian ageing in natural fertility populations. *Maturitas*, *30*(2), 127-136. <https://doi.org/10.1016/S0378-5122(98)00068-1>

Russell, D., Douglas, A. S., & Allan, T. M. (1993). Changing Seasonality of Birth - a Possible Environmental-Effect. *Journal of Epidemiology and Community Health*, *47*(5), 362-367. <https://doi.org/10.1136/jech.47.5.362>

Sandahl, B. (1978). Seasonal Birth Pattern in Sweden in Relation to Birth-Order and Maternal Age. *Acta Obstetricia Et Gynecologica Scandinavica*, *57*(5), 393-396. <https://doi.org/10.3109/00016347809156517>
